# Novelty of Italian Grape Ale (IGA) beer: Influence of the addition of Gamay macerated grape must or dehydrated Aleatico grape pomace on the aromatic profile

**DOI:** 10.1016/j.heliyon.2023.e20422

**Published:** 2023-09-25

**Authors:** Nicola Mastrangelo, Alessandro Bianchi, Stefano Pettinelli, Gregorio Santini, Giorgio Merlani, Andrea Bellincontro, Federico Baris, Fabio Chinnici, Fabio Mencarelli

**Affiliations:** a Department of Agriculture, Food and Environment, University of Pisa, Via Del Borghetto 80, 56124, Pisa, Italy; b Department for Innovation in Biological, Agro-Food and Forest Systems (DIBAF), University of Tuscia, Via De Lellis, 01100, Viterbo, Italy; c Department of Agricultural and Food Sciences, Alma Mater Studiorum, University of Bologna, Viale Fanin 40, Bologna, 40127, Italy

**Keywords:** Italian grape ale, Carbonic maceration, Nitrogen maceration, e-nose, VOCs, Red wine, Beer

## Abstract

A new category of fruit style beer resulting from the addition of grape matrices is named Italian Grape Ale (IGA). In this paper, we report data on an experimental work to produce IGA beers, adding macerated (CO_2_ or N_2_) red Gamay grape must or Aleatico grape pomace resulting from a grape dehydration process. Our hypothesis, that these wine processes can produce volatile organic compounds (VOCs) to characterize these IGA beers which was confirmed by chemical, sensory and aromatic results. IGA beers especially the one with gas-macerated grape musts (IGA-C and IGA-N) showed higher alcohol content than ALE beer (Control) and a higher polyphenol content and antioxidant activity. As regards VOC_S_, IGA beers increased the concentration of some classes (i.e., alcohols, esters, norisoprenoids) and IGA-N was better characterized by specific compounds such as isobutyric acid, phenylacetate, tyrosol, ethyl hydrogen succinate. Finally, E-nose and sensory evaluation discriminated significantly all the IGA beers.

## Graphical abstract

**Figure:**
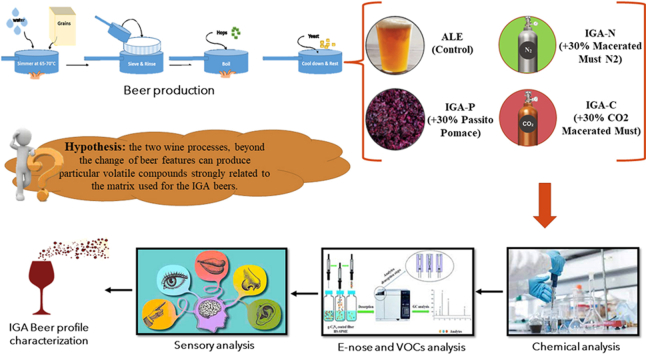

## 1 Introduction

In 2015, a new subcategory of fruit style beer was proposed with the name of Italian Grape Ale (IGA) and introduced in Beer Judge Certification Program (BJCP) as a product resulting from the addition of grape matrices to beer [1]. As reported by Beer Judge Certification Program (2021) [2] the grape content can account for up to 40% of the entire grist. Grape berries or grape must, sometimes boiled before use, can be used in several stages: during boiling or more commonly during primary/secondary fermentation. The grape matrix has not to be fermented or partially fermented, meaning that it has not to contain alcohol.

In the recent years, few papers have been published on IGA beers. De Francesco et al. (2021) [3] analyzed 22 commercial IGA for the volatile organic compounds (VOCs) and sensory evaluation, finding a common features in high ethanol content, low bitterness and low pH, notwithstanding the heterogeneity of the beer process. Castro Marin et al. (2021) [4] added different amounts of must (5, 10 and 20% V/V) from cv. Lambrusco red grapes to a lager wort before primary fermentation and found the addition of must enriched beers in color, acids, phenols, and volatile compounds, giving a sensory evaluation of more complexity to beers. Leni et al. (2023) [1] followed another strategy of addition: they added not only must of white cv. Malvasia di Candia Aromatica but also pomace (10 and 20%); the addition of pomace allowed to significantly enrich final beers in total polyphenols.

Thus, if the addition of must or pomace significantly modifies the beer composition and its aroma, and the extent of this change depends on the amount of the addition, our hypothesis is that the modification of the aroma depends on the type of must or pomace that we add.

Beer is a complex mixture of constituents, brewed from raw materials including water, yeast, malt and hops, and at all stages of the brewing process, VOCs can be produced and released. The volatile fraction can be composed of over 800 different compounds in beer, much more than the number we measured in wine, but only few dozen be flavor-active [[5], [6], [7]]. These compounds belong to several chemical classes, including higher alcohols, esters, fatty acids, carbonyl compounds, sulphur compounds, furanic compounds, monoterpenols, C13-norisoprenoids and volatile phenols [8]. The main VOCs can be derived from barley and hops, as by-products of yeast metabolism, contaminant microorganisms, and from the beer storage [6,9].

When we add grape-derived product (must or pomace), we increase the concentration of some class of VOCs (i.e., alcohols, esters, norisoprenoids) and also the panorama of compounds in each single class [4]. Thus, it depends on the type of must or pomace we add, we can obtain different VOCs panorama and aroma bouquet.

Carbonic maceration is very well known to produce a very famous French wine [10], the Beaujolais while nitrogen maceration is a new proposed process [11].

Italy is the Country with the highest number of Passito wines [12] and we have been studying the dehydration process for several years [13] and overall, in relation to the grape VOCs during the dehydration process [[14], [15], [16]].

Based on the aforementioned our knowledge and current research on grape special processes, we decided to use these grape matrices to produce a novelty IGA beer, completely different from the one marketed and giving the opportunity to wineries, to produce IGA beers with the addition special matrices. Thus, in this paper, we report the data of an experimental work to produce IGA beer, adding must of gas (CO_2_ or N_2_) macerated red cv. Gamay Teinturer grape, typical variety used to produce Beaujolais-type wine, or adding pomace of red cv. Aleatico grape dehydrated, a variety used in Italy to produce sweet wine. Our hypothesis is that the two wine processes which can be used in a winery, beyond the change of beer features overall the color and the structure, can produce particular VOCs which characterize these IGA beers and make them unique.

## 2 Materials and methods

### 2.1 Raw material

The must, used in the production of IGA-C and IGA-N, was obtained from bunches of grapes cv. Gamay Teinturier (*Vitis Vinifera* L.) manually harvested in September 2021, at the Fattoria di Calappiano (Sensi Vigne & Vini, Lamporecchio, Italy). After washing and superficial drying, The grape bunches were placed in two air-tight stainless-steel tanks and saturated with gas (100% CO_2_ or 100% N_2_). All the tests were run at room temperature (22–25 °C). After 7 days of carbonic or nitrogen maceration, the tanks were opened, and the berries were hand removed from the bunch and pressed with a pneumatic press 40 L (Polsinelli Enologia Srl, Isola del Liri, Italy) to obtain the must [11].

The pomace used, on the other hand, was grapes cv Aleatico (*Vitis Vinifera* L.) manually harvested in September 2021, at the Fattoria di Calappiano (Sensi Vigne & Vini, Lamporecchio, Italy). The bunches were placed in perforated plastic crates for the dehydration process in a ventilated tunnel (temperature = 20 ± 2 °C; humidity = 65 ± 3 °C; airflow = 1 m/s; time = 20 days), up to 40% of weight loss [17]. At the end, the bunches were pressed with a pneumatic press 40 L (Polsinelli Enologia Srl, Isola del Liri, Italy), and the pomace, left over from the juice extraction, was used in the production of IGA-P beer.

Beer was produced in spring 2022 and we used the must and the pomace we stored at low temperature (0 ± 2 °C). The chemical characteristics of the two macerated must (CO_2_ and N_2_) and pomace are reported in Table S1.

The ingredients used for beer production are: 10 kg of malt Maris Otter (Muntons, Stowmarket, United Kingdom), 10 kg of malt Pilsner (Weyermann, Bamberg, Germany), 300 g of Willamette hop pellets (BSG Hops, Wapato, United States), 60 g of dry yeast SafAle™ US-05 (Fermentis, Marquette-lez-Lille, France) and water (70 L in mash, 63.6 L in sparge).

### 2.2 Experimentatal setup

For the production of beer, a stainless-steel plant Easy 100 (Polsinelli Enologia Srl, Isola del Liri, Italy) was used following the phases described below: (i) mash: insertion of ground malts at 35 ± 2 °C; (ii) protein rest: 55 °C for 10 min; (iii) mash-in: 65 °C for 50 min; (iv) β–glucan rest: 72 °C for 15 min; (v) mash-out: 78 °C for 5 min; (vi) Sparge with water at 78 °C for the sugar extraction from brewers grains; (vii) boil the beer must obtained for 60 min at 88 ± 2 °C and add hops in 3 portions (100 g) every 20 min; (viii) the must was cooled (23 ± 2 °C) and the yeast was inoculated.

For the fermentation phase, the beer-must was divided into 8 (two each sample) fermenters of 20 L: 1- Control beer (ALE); 2-added 30% V/V of carbonic macerated must (IGA-C); 3-added 30% V/V of nitrogen macerated must (IGA-N); 4- added 30% w/V of pomace (IGA-P). The 30% of addition was chosen in order to provide an intense grape-derived aroma to beer.

The primary fermentation lasted 12 days, 9 days at 18 ± 2 °C and 3 days at 6 °C to allow the precipitation of the solid compounds (lagering). During the bottling phase, 6 g/L of commercial beet sugar were added to start the bottle secondary fermentation which lasted one month.

### 2.3 Chemical analysis

Beer chemical analyses were carried out on four, 0.5 L bottles, two from each fermenter vat, and before of analyses, the beers were degassed by ultrasound. The pomace characterization was conducted on its water extract (1:2 w/V) by ultrasonic bath (Elma TI- H-15, Singen, Germany) for 30 min at 25 °C, while no treatment was used on macerated musts.

The analyses of macerated must and pomace were performed with a calibrated Fourier transform infrared WineScan™ FT 120 (Foss Analytics, Hillerod, Denmark) to determine the following chemical parameters: sugars (g/L hexoses), pH, titratable acidity (tartaric acid g/L), volatile acidity (g/L acetic acid), malic acid (g/L), total anthocyanins (mg/L malvidin), and total polyphenols (mg/L gallic acid). The accuracy of the WineScan™ analyses was validated, periodically, by destructive analyses performed with reference methods as previously reported [18].

The beer chemical analyses of alcohol (% V/V), pH, sugars (g/L hexoses), titratable acidity (g/L lactic acid), volatile acidity (g/L lactic acid), total polyphenols (mg/L gallic acid), total anthocyanins (mg/L malvidin), color according to the SRM (Standard Reference Method) scale and bitterness according to the IBU (International Bitterness Unit) scale, were carried out following the official method of the American Society of Brewing Chemists [19].

Finally, the anti-radical activity of samples (must, pomace and beer) was determined by DPPH and ABTS free radical method as previously reported [20]. The results were expressed as μmol Trolox equivalents (TE) per mL, according to different standard curves of Trolox (in the range 0–200 μmol/L for the DPPH assay, 0.2–1.5 mM range for ABTS assay).

### 2.4 Volatile organic compunds (VOCs)

The analysis of VOCs was performed as reported by López et al. (2002) [21] with minor modifications. In particular, 10 mL sample of beer, degassed by ultrasound, and 100 μL of a 2-octanol solution at 500 mg/L was added as an internal standard. The sample was deposited on a Hypersep Retain Prep (Thermo Fisher Scientific, Milan, Italy) cartridge (60 mg), activated with 2 mL dichloromethane, 2 mL methanol and 2 mL water. The analytes were eluted with 5 mL of dichloromethane, collected in sovirel on the bottom of which 2 g of anhydrous sodium sulfate had been inserted and placed in the freezer overnight. Prior to GC-MS analysis, samples were filtered with a cellulose filter to remove sodium sulfate and concentrated to a final volume of 200 μL under a stream of N_2_.

The GC apparatus consists of a Trace GC ultra-gas chromatograph with a Trace DSQ with quadrupole mass detector (Thermo Fisher Scientific, Milan, Italy) and a Stabilwax DA capillary column (Restek, Bellefonte, PA, USA; 30 m, 0.25 mm i.d., and 0.25 μm film thickness). The carrier gas consists of He with a constant flow of 1.0 mL/min.

GC temperature ramp was programmed as reported by Castro-Marín et al. (2018) [22]: from 45 °C (maintained for 1 min) to 100 °C (maintained for 1 min) at 3 °C/min, then to 240 °C (maintained for 10 min) at 5 °C/min. The injection was performed at 250 °C in splitless mode and the volume injected is 1 μL.

Identification of compounds was carried out by following a triple criterion: (i) by comparing compound mass spectra and retention time with those of pure standards, (ii) matching their respective mass spectra with those present in online libraries Willey and NIST 08, (iii) by comparing linear retention index (LRI) calculated under our analytical conditions with already published LRI calculated on polar columns. Quantification of compounds was carried out via the respective total ion current peak areas after normalization with the area of the internal standard. Calibration curves were obtained by duplicate injections of standard solutions, subjected to the above cited extraction procedure, containing a mixture of commercial standard compounds at concentrations between 0.005 and 30 mg/L, and internal standard at the same concentration as in the samples. The calibration equations for each compound were obtained by plotting the peak area response ratio (target compound/internal standard) versus the corresponding concentration. For compounds lacking reference standards, the calibration curves of standards with similar chemical structure were used. Analyses were done in duplicate (two bottles) and GC-MS parameters were obtained by using Xcalibur software (version 4.1, Thermo Fisher Scientific, Milan, Italy).

### 2.5 E-nose mesurement

The E-nose used consists of an array of eight quartz microbalances (QMB) coated with modified metalloporphyrin (5,10,15,20-tetraphenylporphyrin, TPP), whose selectivity depends on the nature of both the central metal and the peripheral substituent of macrocycles (Mn-TPP, Co-*p*-OCH_3_ TPP, Sn-TPP, Rh-TPP, Co-p-NO_2_, Cr-TPP, Co-TPP, Ru-TPP) [23]. The QMB sensors act as an electromechanical resonator with a natural oscillation frequency ΔF of 20 MHz that change when volatiles interact reversibly with its surface [14].

The protocol for the E-nose detections was adapted from Martínez-García et al. (2021) [24]. In particular, 50 mL of beer sample was first degassed using a partial vacuum with a 500 mL flask and a Venturi pump system for 1 min. Next, 5 mL of degassed beer was placed in a 10 mL vial and tempered to 25 ± 2 °C for 30 min. The VOCs accumulated in the head space of the vial, were transferred to the cells of the E-nose sensor using an air flow for 10 min, which was filtered through a trap filled with anhydrous CaCl_2_. Pure nitrogen (99.99%) at a constant flow of 100 mL/min was used to clean the sensors for 5 min and the signals obtained were considered as a reference for each QMB. Each beer sample was analyzed in three vials and the mean values of each QMB were used for statistical treatment.

### 2.6 Sensory analysis

The beer sensory profile was evaluated by a panel of eight beer experts (brewers and beer sensory teacher). In particular, the sensory sheet (Fig. S1) includes quantitative descriptors (foam stability, foam compactness, color intensity, olfactory intensity, olfactory complexity, floral, fruity, vegetable, malt, effervescence, body, sweet, acid, bitter, astringency, softness, spicy, toasted, persistence) and global quality descriptors (visual attractiveness, olfactory pleasantness, tasting pleasantness). All global quality descriptors refer to an overall judgment, on visual, smell and taste which is the perceived sum of all the previous quantitative descriptors evaluated for that precise sense (visual attractiveness = foam stability, foam compactness and color intensity; olfactory pleasantness = olfactory complexity, floral, fruity, vegetable and malt; tasting pleasantness = effervescence, body, sweet, acid, bitter, astringency, softness, spicy, toasted and persistence). The overall quality index (QI) of beers was calculated, starting from the mean of the global quality descriptors converted on a scale from 0 to 10, as previously reported [25]. The research was conducted according to the ethical guidelines and informed consent was obtained from all participants.

### 2.7 Statistical analysis

One-way ANOVA was run (CoStat, Version 6.451, CoHort Software, Pacific Grove, CA, USA) and Tukey's honestly significant difference (HSD) test with p ≤ 0.05 for multiple comparison, was used for the chemical parameters.

Multivariate statistics computed on normalized VOC measurements were performed by using Matlab R2013a (MathWorks®, Natick, MA, USA), and PLS Toolbox (Eigenvector Research, Inc., Manson, WA, USA). Namely, a principal component analysis (PCA) and a hierarchical cluster analysis (HCA), performed by the Ward's method and reported as dendrogram (E-Nose) or two-way [11].

Sensory analysis results were processed by Big Sensory Soft 2.0 (version 2018). In particular, sensory data were analyzed by two-way ANOVA with panelists and samples as main factors.

## 3 Results and discussion

### 3.1 Chemical characterization of beers

The IGA beers presented higher alcohol content, in particular the one from nitrogen-macerated grapes (Table 1). This result was expected because we added matrices with high sugar content. pH as expected was lower in IGA beer as a result of added matrix which is acid. Volatile acidity was also significantly higher in IGA beer, probably due to the addition of lactic bacteria from the grape matrices which were not sterilized, and this was also observed by De Francesco et al. (2021) [3]. The color as expected was much darker with deep red hue due to the color of added matrices while the IBU was lower in accord with the commercial IGA measured by De Francesco et al. (2021) [3], with an average value around 15.

**Table 1:** Main chemical parameters of beers.

Chemical parameters	Units	ALE	IGA-P	IGA-C	IGA-N
Alcohol	% V/V	7.17 ± 0.11^c^	7.26 ± 0.14 ^bc^	7.52 ± 0.17^b^	8.12 ± 0.12^a^
pH	–	4.12 ± 0.02^a^	3.74 ± 0.03^c^	3.85 ± 0.03^b^	3.77 ± 0.04 ^bc^
Sugars	g/L hexoses	1.98 ± 0.12^c^	3.12 ± 0.11^a^	2.34 ± 0.18^b^	1.74 ± 0.16^c^
Titratable acidity	g/L lactic acid	1.89 ± 0.19^d^	3.84 ± 0.20^a^	2.79 ± 0.13^c^	3.32 ± 0.19^b^
Volatile acidity	g/L acetic acid	0.21 ± 0.02^c^	0.48 ± 0.05^a^	0.32 ± 0.04^b^	0.35 ± 0.03^b^
Color (SRM)	–	7.2 ± 1.3^d^	17.3 ± 1.6^c^	39.9 ± 1.1^b^	44.4 ± 1.2^a^
IBU	–	26.5 ± 1.1^a^	15.3 ± 0.9^d^	18.8 ± 0.7^b^	17.5 ± 0.6^c^
Total polyphenols	mg/L gallic acid	405 ± 25^d^	541 ± 31^c^	1080 ± 19^b^	1270 ± 21^a^
Total anthocyanins	mg/L malvidin	n.d.	62 ± 9^c^	632 ± 20^b^	701 ± 15^a^
ABTS	μmol TE/mL	0.36 ± 0.02^d^	0.50 ± 0.05^c^	1.03 ± 0.03^b^	1.17 ± 0.04^a^
DPPH	μmol TE/mL	0.19 ± 0.02^d^	0.24 ± 0.02^c^	0.59 ± 0.04^b^	0.69 ± 0.03^a^

Data are the mean of 4 bottle analyses. Different letters in each row refer to significant differences (Tukey, *p* ≤ 0.05). n.d. = not detected.

As expected the total polyphenols and total anthocyanins were significantly higher overall in IGA with gas-macerated must (IGA-C and IGA-N) because the grape variety used is rich in these compounds; these values affected positively the antioxidant activities of beers increasing it by 2–3 folds.

### 3.2 Aromatic profile of beers

As in all fermented beverages, apart from ethanol and carbon dioxide, which are the main products of fermentation, there are also classes of VOCs in beer which are formed as products secondary to fermentation and characterize its quality. Olaniran et al. (2017) [8] reported what they define as “Flavour-active volatile compounds in beer”. Higher alcohols and esters represent the most important classes as in wine, being the result of fermentation by yeasts. Among the esters, 1/3 is represented by ethyl acetate [26] which if in low concentration provides solvent nuance. The threshold concentration of ethyl acetate in beer is 30 mg/L, but for lager-type beers the recommended concentration is < 5 mg/L. The intense "fruity" aroma caused by isoamyl acetate and 2-phenylethyl acetate is found at concentrations >2 and 3.8 mg/L, respectively. Ethyl hexanoate has a low threshold concentration of 0.005 mg/L, ethyl octanoate at 0.5 mg/L and ethyl decanoate at 1.5 mg/L [27]. When these concentrations exceed their thresholds, they impart an unwanted flavor to the beer. Other compound classes are ketones, volatile phenols, acids which to a lesser extent add complexity to the aroma of beer. The IGA beers of this experimentation have a much more complex aromatic profile in fact, in addition to alcohols, to a significantly higher extent than the other classes, and esters, we have quantified the class of acids in high concentration, even higher than esters in terms of concentration (Table 2).

**Table 2:** Volatile Organic Compounds (VOCs) in beers.

VOCs (μg/L)	ALE	IGA-P	IGA-C	IGA-N
Ethyl butyrate	98.10 ± 3.90^b^	40.75 ± 2.41^c^	201.27 ± 3.42^a^	203.96 ± 4.52^a^
Ethyl isovalerate	n.d.	8.56 ± 0.58^a^	n.d.	n.d.
Isoamyl acetate	1096.38 ± 73.25^a^	143.79 ± 12.5^b^	1212.93 ± 111.95^a^	1115.68 ± 95.89^a^
Ethyl esanoate	231.61 ± 5.66^b^	147.81 ± 12.84^c^	495.95 ± 31.73^a^	490.78 ± 35.94^a^
Hexyl acetate	5.05 ± 0.84^a^	n.d.	4.35 ± 0.88^a^	n.d.
Ethyl heptanoate	20.81 ± 0.21^b^	24.82 ± 3.58^a^	23.92 ± 3.12^a^	23.08 ± 2.85^a^
Ethyl lactate	106.68 ± 8.57 ^ab^	122.40 ± 21.26^a^	98.59 ± 8.02^b^	103.35 ± 4.66^b^
Ethyl octanoate	368.65 ± 39.32^b^	126.73 ± 17.47^c^	596.33 ± 77.85^a^	491.09 ± 67.72^a^
Ethyl decanoate	51.80 ± 6.77^b^	52.46 ± 5.43^b^	59.52 ± 4.07^b^	85.50 ± 6.01^a^
Diethyl succinate	46.13 ± 3.11^d^	72.40 ± 9.75^c^	137.39 ± 3.84^b^	155.93 ± 8.82^a^
Ethyl 9-decenoate	68.98 ± 6.52^a^	n.d.	41.86 ± 3.33^b^	30.93 ± 5.94^c^
1,3-Diacetoxypropane	79.68 ± 5.15^b^	123.21 ± 17.91^a^	79.37 ± 12.67^b^	82.92 ± 9.65^b^
Methyl salicylate	n.d.	5.70 ± 0.65^a^	n.d.	n.d.
Ethyl phenylacetate	8.87 ± 0.34^b^	29.76 ± 0.88^a^	8.84 ± 0.29^b^	8.72 ± 1.33^b^
Etil-4-idrossibutanoato	128.16 ± 7.45^b^	53.35 ± 5.64^c^	166.85 ± 22.89^a^	175.48 ± 26.5^a^
2-Feniletilacetato	660.83 ± 14.59^a^	69.70 ± 3.55^c^	461.14 ± 19.86^b^	412.64 ± 38.44^b^
Ethyl 3-phenylpropionate	16.26 ± 1.31^a^	6.67 ± 0.21^c^	10.80 ± 0.84^b^	11.81 ± 0.25^b^
Ethyl cinnamate	14.07 ± 1.23^b^	n.d.	16.26 ± 0.69^a^	15.51 ± 1.33 ^ab^
Ethyl 2-hydroxy-3-phenylpropanoate	150.14 ± 1.05^b^	155.57 ± 6.47^b^	152.33 ± 5.78^b^	221.20 ± 2.89^a^
Ethyl hydrogen succinate	110.11 ± 16.22^d^	281.50 ± 22.17^c^	357.43 ± 17.11^b^	436.38 ± 35.27^a^
Methyl vanillate	n.d.	12.51 ± 3.37^c^	131.50 ± 4.72^b^	160.97 ± 6.62^a^
Ethyl vanillate	n.d.	85.02 ± 11.06^a^	30.95 ± 4.56^c^	42.27 ± 5.37^b^
Total Esters	3265.28 ± 8.48^b^	1564.97 ± 6.85^c^	4290.09 ± 15.52^a^	4270.92 ± 28.33^a^
1-Propanol	36.30 ± 4.05^b^	19.40 ± 3.42^c^	55.32 ± 5.32^a^	49.02 ± 4.44^a^
Isobutanol	897.09 ± 46.48^a^	563.49 ± 35.72^c^	877.71 ± 47.52^a^	811.35 ± 19.85^b^
*trans*-3-Penten-2-ol	14.04 ± 1.09^a^	12.43 ± 1.12^a^	13.14 ± 1.51^a^	13.91 ± 1.98^a^
Isoamyl alcohol	10228.71 ± 589.25^a^	6846.38 ± 180.36^b^	10044.06 ± 786.55^a^	11313.10 ± 664.48^a^
1-Pentanol	10.30 ± 1.26 ^ab^	12.01 ± 1.04^a^	9.30 ± 0.89^b^	9.18 ± 1.02^b^
4-Metil-1-pentanol	2.58 ± 1.37^c^	2.25 ± 1.66^c^	7.17 ± 0.87^b^	10.79 ± 1.55^a^
3-Metil-1-pentanol	n.d.	n.d.	15.42 ± 0.77^b^	20.86 ± 0.44^a^
n-Hexanol	62.92 ± 1.51^d^	306.18 ± 19.86^a^	88.94 ± 1.24^c^	122.56 ± 10.03^b^
3-Ethoxy-1-propanol	15.23 ± 1.87^b^	14.64 ± 2.89^b^	16.94 ± 3.84^b^	24.20 ± 2.54^a^
*cis*-3-Hexen-1-ol	6.24 ± 0.56^b^	13.91 ± 0.99^a^	3.29 ± 0.83^c^	4.06 ± 0.81^c^
2-etylhexanol	21.96 ± 2.79^b^	48.62 ± 3.11^a^	10.97 ± 1.64^c^	10.86 ± 0.99^c^
3-nonanol	7.74 ± 1.15^b^	12.20 ± 0.99^a^	8.44 ± 0.89^b^	7.44 ± 1.85^b^
2-nonanol	13.65 ± 0.28^a^	11.00 ± 1.04^b^	11.04 ± 1.37^b^	8.29 ± 1.86^c^
2,3-Butanediol	699.85 ± 99.07^c^	547.88 ± 47.56^d^	1151.36 ± 117.52^a^	970.65 ± 35.54^b^
1-octanol	19.68 ± 1.02^b^	24.89 ± 1.86^a^	25.35 ± 2.89^a^	26.61 ± 3.21^a^
2-3-butanediol	478.98 ± 61.78 ^ab^	547.88 ± 80.61^a^	414.43 ± 24.52^b^	299.80 ± 16.85^c^
1-2-propanediolo	21.37 ± 2.11^c^	33.24 ± 5.14^b^	47.79 ± 4.56^a^	37.35 ± 2.92^b^
1-Methoxy-2-butanol	8.69 ± 0.76^a^	7.90 ± 0.62 ^ab^	7.74 ± 0.58 ^bc^	6.62 ± 0.54^c^
Methionol	48.76 ± 3.96^c^	72.81 ± 2.69^b^	128.17 ± 10.51^a^	125.26 ± 5.86^a^
1-Phenylethanol	n.d.	10.54 ± 0.47^a^	n.d.	5.38 ± 0.69^b^
Benzyl alcohol	28.19 ± 2.11^c^	78.69 ± 3.98^a^	49.23 ± 1.25^b^	42.39 ± 6.85^b^
Phenethyl alcohol	31633.00 ± 1231.31^a^	17394.88 ± 782.39^d^	21546.33 ± 1765.15^c^	26830.70 ± 2238.26^b^
Benzene propanol	n.d.	10.41 ± 0.28^c^	14.53 ± 0.26^b^	26.84 ± 0.44^a^
2-Phenoxyethanol	10.07 ± 0.86^b^	17.08 ± 2.46^a^	n.d.	8.89 ± 0.37^c^
Total Alcohols	44265.35 ± 69.33^a^	26608.71 ± 87.55^d^	34546.67 ± 133.22^c^	40786.11 ± 142.59^b^
Acetic acid	328.24 ± 89.56^d^	6834.58 ± 508.29^a^	798.57 ± 128.24^c^	1068.59 ± 214.73^b^
Propanoic acid	14.83 ± 2.99^b^	22.84 ± 3.14^a^	8.01 ± 1.13^c^	13.10 ± 2.04^b^
Isobutyric acid	570.66 ± 33.98^a^	362.53 ± 58.02^b^	159.25 ± 43.47^c^	151.85 ± 33.27^c^
Butanoic acid	44.79 ± 5.21^b^	28.73 ± 4.62^c^	60.19 ± 6.72^a^	63.51 ± 2.43^a^
Valeric acid	505.15 ± 36.92^b^	956.54 ± 122.07^a^	295.70 ± 50.20^b^	332.17 ± 39.38^b^
Hexanoic acid	1872.67 ± 54.44^c^	1097.24 ± 40.85^d^	2087.37 ± 22.41^b^	2138.73 ± 27.56^a^
3-Hexenoic acid	259.17 ± 17.04^a^	199.84 ± 10.25^b^	164.30 ± 6.21^c^	161.97 ± 8.56^c^
E)-2-Hexenoic acid	n.d.	79.20 ± 10.22^a^	n.d.	n.d.
Caprylic acid	3940.54 ± 71.22^b^	1292.75 ± 46.15^c^	4074.37 ± 162.58 ^ab^	4218.19 ± 183.75^a^
Nonanoic acid	28.56 ± 1.36^a^	24.68 ± 1.71^b^	17.28 ± 0.59^d^	20.92 ± 1.60^c^
Capric acid	746.57 ± 43.05^a^	374.22 ± 35.24^c^	406.63 ± 48.21^c^	650.25 ± 34.83^b^
9-decenoic acid	869.85 ± 36.08^a^	52.38 ± 7.02^d^	332.81 ± 6.26^c^	365.53 ± 14.52^b^
Benzoic acid	223.13 ± 9.61^c^	381.51 ± 36.22^a^	229.51 ± 6.23^c^	244.50 ± 18.62 ^bc^
Lauric Acid	84.87 7.58^b^	83.76 ± 8.83^b^	103.09 ± 6.12^a^	117.65 ± 18.74^a^
Benzene acetic acid	573.31 ± 33.31^b^	2150.50 ± 143.04^a^	368.03 ± 18.12^d^	455.31 ± 37.61^c^
Phenyl propionic acid	46.02 ± 2.37^a^	33.96 ± 2.36^c^	39.22 ± 1.03^b^	47.56 ± 3.66^a^
Myristic acid	52.38 2.79^b^	63.79 ± 3.55^a^	53.34 ± 4.62^b^	37.36 ± 1.81^c^
Palmitic acid	328.72 ± 20.68^a^	372.74 ± 32.35^a^	267.02 ± 22.35^b^	361.34 ± 45.92^a^
Stearic acid	218.28 ± 17.69^a^	141.20 ± 13.45^b^	89.74 ± 7.86^c^	128.46 ± 11.57^b^
Total Acids	10707.74 ± 28.35^b^	14553.11 ± 85.42^a^	9554.54 ± 65.98^c^	10577.08 ± 78.56^b^
2,7-Dimethyl-2,6-octadiene	432.69 ± 17.47^a^	167.06 ± 11.02^c^	203.26 ± 8.56^b^	218.17 ± 13.06^b^
6-Methyl-5-hepten-2-one	5.81 ± 0.53^a^	5.84 ± 0.84^a^	4.33 ± 0.35^b^	3.60 ± 0.33^c^
Linalool oxide	4.05 ± 0.11^b^	11.01 ± 1.82^a^	4.17 ± 0.16^b^	3.29 ± 0.22^c^
Pinocamphone	11.91 ± 1.32^b^	16.36 ± 2.35^a^	10.88 ± 0.87 ^bc^	9.16 ± 0.49^c^
β-Linalool	342.99 ± 9.89^a^	353.49 ± 12.41^a^	248.38 ± 16.57^b^	235.94 ± 14.95^b^
Terpinen-4-ol	5.48 ± 1.01^c^	4.41 ± 1.46^c^	8.72 ± 0.99^b^	13.05 ± 2.57^a^
Myrcenol	8.25 ± 0.53 ^ab^	8.44 ± 0.77^a^	8.49 ± 0.25^a^	7.42 ± 0.83^b^
α-Terpineol	62.32 ± 2.02^b^	113.18 ± 6.11^a^	55.56 ± 2.77^c^	53.12 ± 3.92^c^
β-Citronellol	53.20 ± 1.06^b^	114.38 ± 5.09^a^	27.33 ± 3.54^c^	33.70 ± 3.79^c^
cis-Geraniol	18.15 ± 1.89^b^	51.51 ± 0.94^a^	12.45 ± 1.14^c^	11.23 ± 2.42^c^
2,6-Dimethyl-3,7-octadiene-2,6-diol	n.d.	28.01 ± 1.61^a^	n.d.	n.d.
Cubenol	1.14 ± 0.06^a^	0.31 ± 0.06^c^	0.72 ± 0.12^b^	1.00 ± 0.08^a^
α-Bisabolol	28.69 ± 1.36^a^	13.63 ± 2.45^c^	16.47 ± 0.98^c^	19.23 ± 1.02^b^
Geranic acid	49.27 ± 7.91^b^	343.68 ± 21.13^a^	22.48 ± 1.82^d^	26.42 ± 1.42^c^
Farnesol	40.23 ± 1.10^b^	35.32 ± 2.24^c^	49.89 ± 1.70^a^	52.94 ± 2.76^a^
Total Terpenes	1064.18 ± 30.02^b^	1266.63 ± 60.12^a^	673.13 ± 17.35^c^	688.27 ± 24.58^c^
2,4-Dihydroxy-2,5-dimethyl-3(2H)-furan-3-one	10.20 ± 1.74^c^	17.56 ± 1.78^a^	14.09 ± 2.52 ^ab^	13.84 ± 2.04^b^
Dihydro-3-methyl 2(3H)-furanone	n.d.	11.83 ± 0.19^a^	n.d.	6.93 ± 0.95^b^
Furfuryl alcohol	64.18 ± 2.22^a^	59.54 ± 1.58^b^	62.47 ± 3.11 ^ab^	49.70 ± 2.18^c^
5-Hydroxymethyl-dihydro-furan-2-one	6.44 ± 0.34^b^	13.96 ± 0.79^a^	6.37 ± 0.26^b^	5.39 ± 0.56^c^
2,3-Dihydro-5-hydroxy-6-methyl-4H-pyran-4-one	46.69 ± 1.98^a^	7.81 ± 0.36^d^	36.97 ± 0.84^b^	29.97 ± 2.02^c^
2H-Pyran-2,6(3H)-dione	n.d.	n.d.	39.97 ± 1.82^a^	38.12 ± 1.56^a^
2,3-Dihydro-1-benzofuran	47.56 ± 3.16^a^	40.49 ± 1.92^b^	29.65 ± 0.62^d^	33.22 ± 1.09^c^
5-Hydroxymetylfurfural	36.21 ± 2.18^a^	35.97 ± 3.32^a^	18.58 ± 2.14^b^	15.25 ± 3.76^c^
Total Furans	211.28 ± 4.10^a^	187.16 ± 3.81^b^	208.10 ± 2.53^a^	192.42 ± 2.98^b^
*p*-Guaiacol	n.d.	4.88 ± 0.63^a^	5.51 ± 1.36^a^	4.88 ± 0.89^a^
Isoeugenol	15.61 ± 1.75 ^bc^	24.37 ± 2.26^a^	13.51 ± 2.45^c^	17.76 ± 1.23^b^
4-Ethylphenol	n.d.	n.d.	7.16 ± 0.79^b^	9.53 ± 1.09^a^
2-Methoxy-4-vinylphenol	235.56 ± 15.91^a^	134.72 ± 9.98^b^	85.08 ± 5.87^c^	79.91 ± 4.28^c^
Propiovanillone	30.65 ± 1.88^b^	67.00 ± 8.41^a^	23.71 ± 3.86^c^	26.97 ± 1.73^c^
trans-Cinnamic acid	114.99 ± 13.44^b^	n.d.	148.64 ± 13.02^a^	147.05 ± 14.52^a^
Tyrosol	954.99 ± 80.22^a^	324.29 ± 82.42^c^	781.08 ± 34.16^b^	1168.62 ± 183.07^a^
Total Phenols	1351.80 ± 23.24^b^	555.26 ± 25.43^d^	1064.69 ± 18.90^c^	1454.72 ± 43.75^a^
Acetoin	65.02 ± 12.02^c^	39.64 ± 6.38^d^	189.42 ± 19.22^b^	387.70 ± 33.51^a^
3-Hydroxy-3-methyl-2-butanone	18.35 ± 1.06^a^	16.53 ± 0.78^b^	19.14 ± 1.28^a^	16.84 ± 0.39^b^
γ-Butyrolactone	6.30 ± 0.88^c^	22.10 ± 1.38^b^	31.90 ± 2.67^a^	33.08 ± 1.90^a^
γ-Nonalactone	135.94 ± 6.71^b^	186.02 ± 14.20^a^	110.09 ± 11.31^c^	106.52 ± 9.36^c^
Total Ketones	225.61 ± 2.96^d^	264.29 ± 5.18^c^	350.55 ± 9.19^b^	544.14 ± 16.01^a^
β-Damascenone	5.74 ± 0.45^c^	7.56 ± 0.18^b^	7.62 ± 0.35^b^	8.58 ± 0.28^a^
3-Hydroxy-β-damascone	21.00 ± 1.00^b^	27.88 ± 3.56^a^	16.68 ± 1.42^c^	18.44 ± 0.89^c^
Total Norisoprenoids	26.74 ± 0.75^b^	35.44 ± 1.87^a^	24.30 ± 0.88^c^	27.02 ± 0.60^b^
n-Nonanal	4.23 ± 0.48^a^	3.54 ± 0.18^b^	3.52 ± 0.25^b^	2.86 ± 0.29^c^
Total Aldehydes	4.23 ± 0.48^a^	3.54 ± 0.18^b^	3.52 ± 0.25^b^	2.86 ± 0.29^c^
*cis*-5-Hydroxy-2-methyl-1,3-dioxane	3.65 ± 1.69^c^	3.97 ± 1.56^c^	10.81 ± 1.66^a^	8.33 ± 0.54^b^
*trans*-4-hydroxymethyl-2-methyl-1,3-dioxolane	15.35 ± 1.32^c^	14.11 ± 1.22^c^	19.01 ± 1.67^b^	26.88 ± 2.44^a^
N-(3-Methylbutyl)acetamide	n.d.	5.29 ± 1.39^c^	12.93 ± 1.84^b^	18.68 ± 2.73^a^
2-Acetylpyrrole	46.67 ± 1.31^b^	53.61 ± 2.51^a^	24.24 ± 2.56^c^	27.42 ± 1.83^c^
Total Others	65.67 ± 1.44^c^	76.98 ± 1.67^b^	66.99 ± 1.93^c^	81.31 ± 1.76^a^

Data are the mean of 2 bottle analyses. Different letters in each row refer to significant differences (Tukey, *p* ≤ 0.05). n.d. = not detected.

Other classes in lower concentrations but important from an aromatic point of view are terpenes, furans, ketones, nor-isoprenoids. The highest concentration of esters was measured in the IGA-C (4290 μg/L) and IGA-N (4270 μg/L) followed by the ALE (3265 μg/L) (Table 2). In beer with pomace, the ester content is somewhat reduced (1565 μg/L). If we look at the panorama of esters that characterize IGAs with gas macerated grape musts, we observe isoamyl acetate (intense fruity notes, banana, apple) in concentrations higher than mg/L, therefore above the olfactory threshold [8]. The other esters in high concentration are ethyl caproate and ethyl caprylate (sour apple hints), esters therefore formed from ethyl alcohol with fatty acids (hexanoic and octanoic acid, respectively) of the cell membrane. Phenylacetate (hints of honey, rose, ripe apple) is also high in concentration but especially in the ALE. These high concentrations of fruity scents in the IGA must beers are due to the refermentation in bottles in which the presence of the must and therefore of a part of the grape sugars, has favored the growth of the yeasts and therefore the formation of a quantity of fatty acids; moreover, a certain number of fatty acids also came from the wort. The same cannot be said for the pomace of dehydrated grapes which, exhausted by the must removal, have not favored the yeasts in the formation of the microbial mass and therefore of the membrane, nor have they added membrane fatty acids. The presence in IGA beers of methyl and ethyl vanillate is interesting, certainly deriving from grapes. Higher alcohols, as mentioned, are in greater concentration in ALE beer followed by IGA-N and IGA-C (Table 2). Phenethyl alcohol (phenylethanol) together with isoamyl alcohol (3-methylbutanol, alcohol scent) are the alcohols in much higher concentration than other alcohols in ALE beer and macerated IGAs. In particular, phenylethanol (rose, sweetish, scented) is higher in Ale beer. These alcohols are formed by the Ehrlich pathway of amino acids degradation and therefore are formed by the fermenting yeasts. Where the fermentation has been more intense there is generally more formation and during the grape maceration in gas, these alcohols are particularly formed [11]. In the macerated IGAs, a high concentration of 2,3-butanediol is probably due to the intervention of malolactic fermentation. Particular is the presence of hexanol in a higher content in the IGA-P; this is due to the high concentration of C6 compounds (green grass taste) in the exhausted skins of dehydrated grapes which have had an intense oxidation of membrane lipids [28]. In the class of acids, on the other hand, the highest concentration was in the IGA-P, in particular due to the presence of acetic acid, significantly higher than in other beers. This high concentration is probably due to the presence of lactic rather than acetic bacteria present on the skins of the dehydrated grapes; we must remember that these types of grapes already have a higher than normal concentration of acetic acid which then increases with fermentation [29]. However, the other IGA beers also had higher concentrations than the Ale but not so high as IGA-P, however highlighting that the addition of must or pomace has to be adequately dosed and above all, from a microbial point of view, they must be as clean as possible. In our case, the high quantity of addition, favored anomalous fermentations, in particular the malolactic fermentation could have started which favored the formation of 2,3-butanediol and the formation of acetic acid. An acid in high concentration in macerated IGAs is hexanoic acid, a membrane fatty acid, probably the sum of the presence of yeasts and macerated grapes which generally tends to form the ethyl hexanoic ester (aniseed, apple scent) which we have not detected. As expected, the IGA-P being from Aleatico grapes, has the highest concentration of terpenes, in particular terpineol, geraniol and citronellol typical of Aleatico [30]. However, the terpenes with the highest content were linalool and dimethyl-octadiene (Table 2).

In the class of furans, no significant differences were observed apart from finding compounds such as HMF (5-hydroxymethylfurfural), typically formed during the brewing process. Phenols and ketones are two classes in minimum concentration but can give unpleasant aromas to the beer; the highest concentration has been measured in IGA-N beer due to the presence of tyrosol, an alcohol that is formed from tyrosine during fermentation. A high concentration of 2-methoxy-4-vinyl-phenol (compound not always desired due to unpleasant aroma) in ALE beer and high level of *trans*-cinnamic in IGA, with hints of cinnamon, were measured. Finally, among the norisoprenoids, it's worth noting the highest presence of damascenone (coconut aroma, tropical fruit) in IGA beers.

**Fig. 1:**
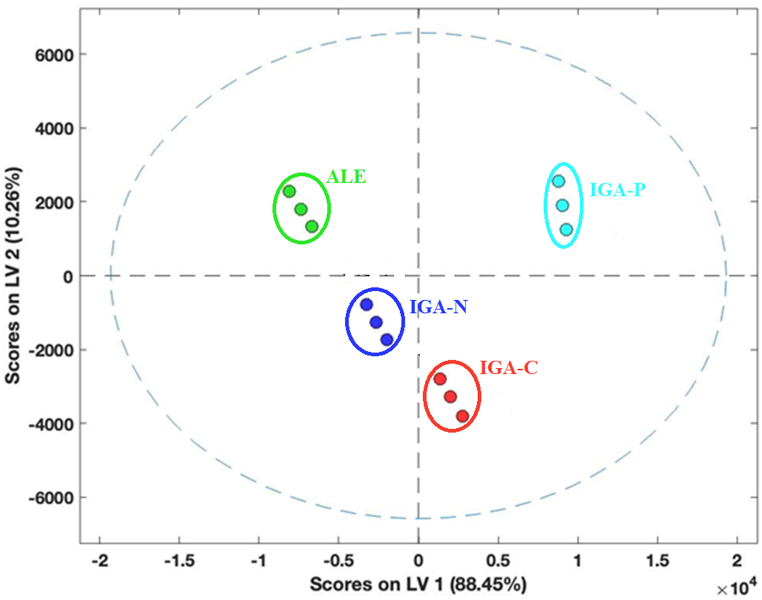
Scoreplot PLS-DA of E-nose readings (three bottles).

From this examination, therefore, a marked aromatic complexity of the IGA beers appears and among these, particularly those with gas macerated grape must. E-nose data elaboration by PLS-DA (Fig. 1), revealed that IGA-P is the most different from the other three. IGA-P is discriminated from IGA-C, IGA-N, and ALE on LV1 (latent variable 1; 88.5% of variance explained), while IGA-C and IGA-N, are less distant from each other (more similar) because they are segregated on LV2 (latent variable 2; 10.2% variance explained) but also on LV1.

**Fig. 2:**
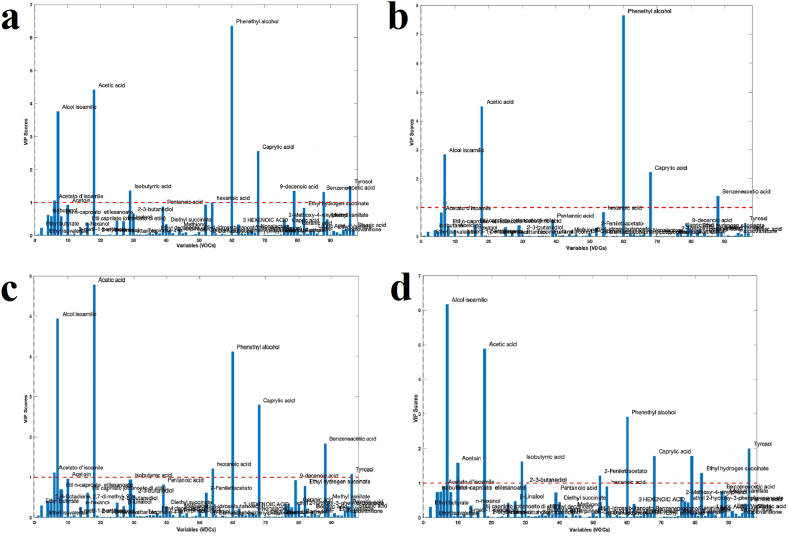
Variables Impact Projections (VIP) scores. The significance threshold is indicated by the dashed red line: (**a**) ALE, (**b**) IGA-P, (**c**) IGA-C, (**d**) IGA-N.

The VIPs are the variables impact projections, i.e., the impact of the variables (VOCs) on the differentiation of the beers from each other (Fig. 2a, b, c, d). Threshold 1 (the red hatch) is the threshold of significance. We can therefore observe that, although the ANOVA had given 97 significantly different VOCs, those that influence the differentiation of beers are few. The ones that characterize each group of beers, are very significant and mark each beer. We notice how in IGA-N beer the VIPs are greater than in other beers and some VOCs are emphasized such as isobutyric acid, phenylacetate, tyrosol, ethyl hydrogen succinate (Fig. 2d). The IGA-C beer has hexanoic acid as a characterizing factor compared to the others (Fig. 2c). The IGA-P is the least rich in characterizing compounds (Fig. 2b). However, the latter is the one that has phenyl-ethanol (phenethyl alcohol) as the most influencing factor compared to the others (higher VIP value) as well as the ALE beer (Fig. 2a). In gas macerated IGAs, isoamyl alcohol is more pronounced than in the other two. Caprylic acid is constant in values, across all four beers.

### 3.3 Sensory evaluation

**Fig. 3:**
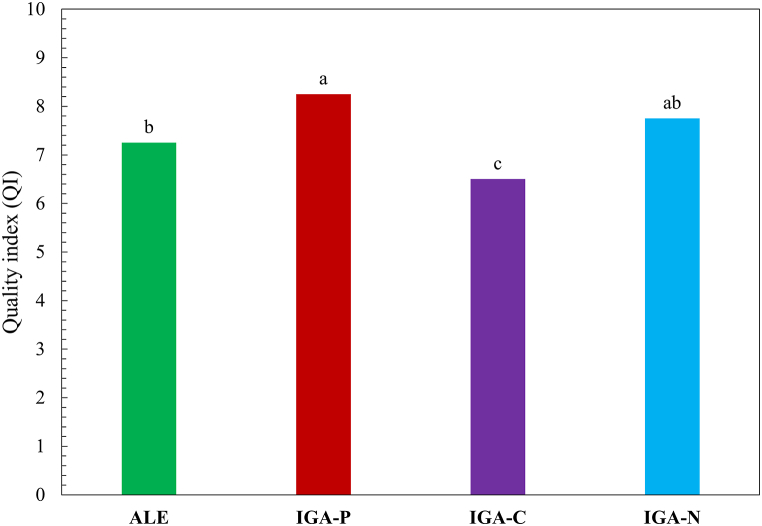
Overall Quality index (QI) of different types of beer. Different letters indicate significant differences among samples (*p* ≤ 0.05).

The global quality level of a product is fundamental in determining its sensory quality and overall pleasantness of the product. As showed in Fig. 3, all the samples reached a positive quality index (QI > 6). In particular, the highest index was attributed to IGA-P and IGA-N beers, followed by ALE (control beer). The lowest value was found for the IGA-C, which was the least preferred by the panel test.

**Fig. 4:**
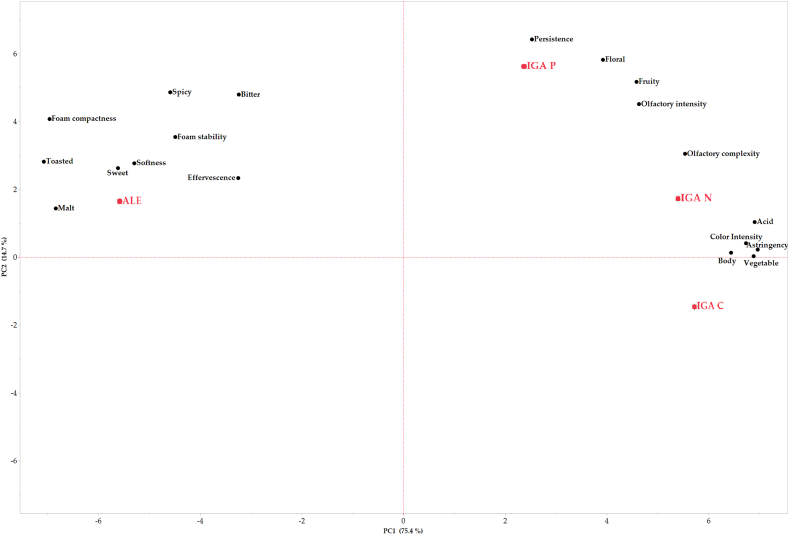
Principal component analysis (PCA) of quantitative descriptors of sensory evaluation of beers.

Going into detail of the parameters that have characterized the various beers (Fig. 4), as expected, ALE is characterized mainly by malty and toasted notes, bitterness and compactness and stability of the foam.

On the other hand, IGA-P, presents a more intense, complex and persistent perceived olfactory profile, with floral and fruity notes, due to the presence of terpenes which characterize the Aleatico grape variety. Finally, the two beers with macerated musts (IGA-C and IGA-N), are characterized by a greater acidity, astringency and body of the beer deriving from typically compounds (tartaric acid, polyphenols and anthocyanins) present in the macerated musts, which also affect color (intense purple red). Both presented a lower stability and compactness of the foam confirming what observed by DeFrancesco et al. (2021) [3], which reported that IGAs had low protein content due to the addition of grape must (a dilution effect), and low hop α-acid, according to reduced bitterness. They found also a significant negative correlation between foam quality and percentage of added grape must.

## 4 Conclusions

The addition of must of gas-macerated grape or pomace of dehydrated grape represent a novelty to produce IGA beer. The strength of these addition is to produce beers with high polyphenols and antioxidant activities, and a more complex aroma, in particular for the IGA beer with nitrogen maceration must. The addition of Aleatico dehydrated grape pomace provided less polyphenol content but a prominent aromatic nuance (floral) due to the use of Aleatico grape. The main problem, a limitation, with these beers has been a somewhat high volatile acidity due to a bacteria-rich grape material (must and pomace), and the scarce presence and instability of the foam due, probably, to the high amount of added grape matrices. Future studies should focus on finding, for each type of raw material, the quantity that exalts its characteristics and peculiarities without altering too much the beer characteristic.

## Author contribution statement

Nicola Mastrangelo: Performed the experiments; Analyzed and interpreted the data; Wrote the paper.

Alessandro Bianchi: Conceived and designed the experiments; Performed the experiments; Analyzed and interpreted the data; Wrote the paper.

Stefano Pettinelli, Gregorio Santini, Giorgio Merlani, Federico Baris: Performed the experiments; Analyzed and interpreted the data.

Andrea Bellincontro, Fabio Chinnici: Contributed reagents, materials, analysis tools or data; Wrote the paper.

Fabio Mencarelli: Conceived and designed the experiments; Contributed reagents, materials, analysis tools or data; Wrote the paper.

## Declaration: ethics statement

The research obtained the approval of the Ethics Committee of the University of Pisa (protocol n. 0088081/2020). The research was conducted according to the ethical guidelines and informed consent was obtained from all participants.

## Data availability statement

Data will be made available on request.

## Declaration of competing interest

The authors declare that they have no known competing financial interests or personal relationships that could have appeared to influence the work reported in this paper.
