# Supplementary material for: Novelty of Italian Grape Ale (IGA) beer: Influence of the addition of Gamay macerated grape must or dehydrated Aleatico grape pomace on the aromatic profile
Source: Heliyon. 2023 Sep 25;9(10):e20422. doi: 10.1016/j.heliyon.2023.e20422 (PMC10539957; doi:10.1016/j.heliyon.2023.e20422)
Supplement: Multimedia component 1 [file mmc1.docx]

**Table S1.** Chemical characteristics of two macerated must (CO_2_ and N_2_) and pomace used in the production of IGA beer.

| **Chemical parameters** | **Units** | **Must CO_2_** | **Must N_2_** | **Aleatico Pomace** |
| --- | --- | --- | --- | --- |
| Alcohol | % V/V | 0.82 ± 0.04 | 1.20 ± 0.08 | 0.04 ± 0.03 |
| pH | - | 3.37 ± 0.02 | 3.36 ± 0.03 | 3.57 ± 0.02 |
| Titratable acidity | g/L tartaric acid | 5.54 ± 0.11 | 5.73 ± 0.13 | 2.89 ± 0.17 |
| Malic acid | g/L | 1.26 ± 0.07 | 1.14 ± 0.08 | 0.14 ± 0.02 |
| Volatile acidity | g/L acetic acid | 0.30 ± 0.02 | 0.28 ± 0.03 | 0.47 ± 0.03 |
| Sugars | g/L hexoses | 190.3 ± 1.2 | 187.8 ± 1.4 | 18.3 ± 1.1 |
| Total polyphenols | mg/L gallic acid | 2937 ± 71 | 3069 ± 92 | 1148 ± 58 |
| Total anthocyanins | mg/L malvidin | 1556 ± 67 | 1601 ± 84 | 384 ± 41 |
| ABTS | μmol TE/mL | 2.51 ± 0.01 | 2.63 ± 0.02 | 1.12 ± 0.02 |
| DPPH | μmol TE/mL | 1.24 ± 0.02 | 1.29 ± 0.01 | 0.61 ± 0.01 |

Data are the mean (± SD) of three readings from three different samples.


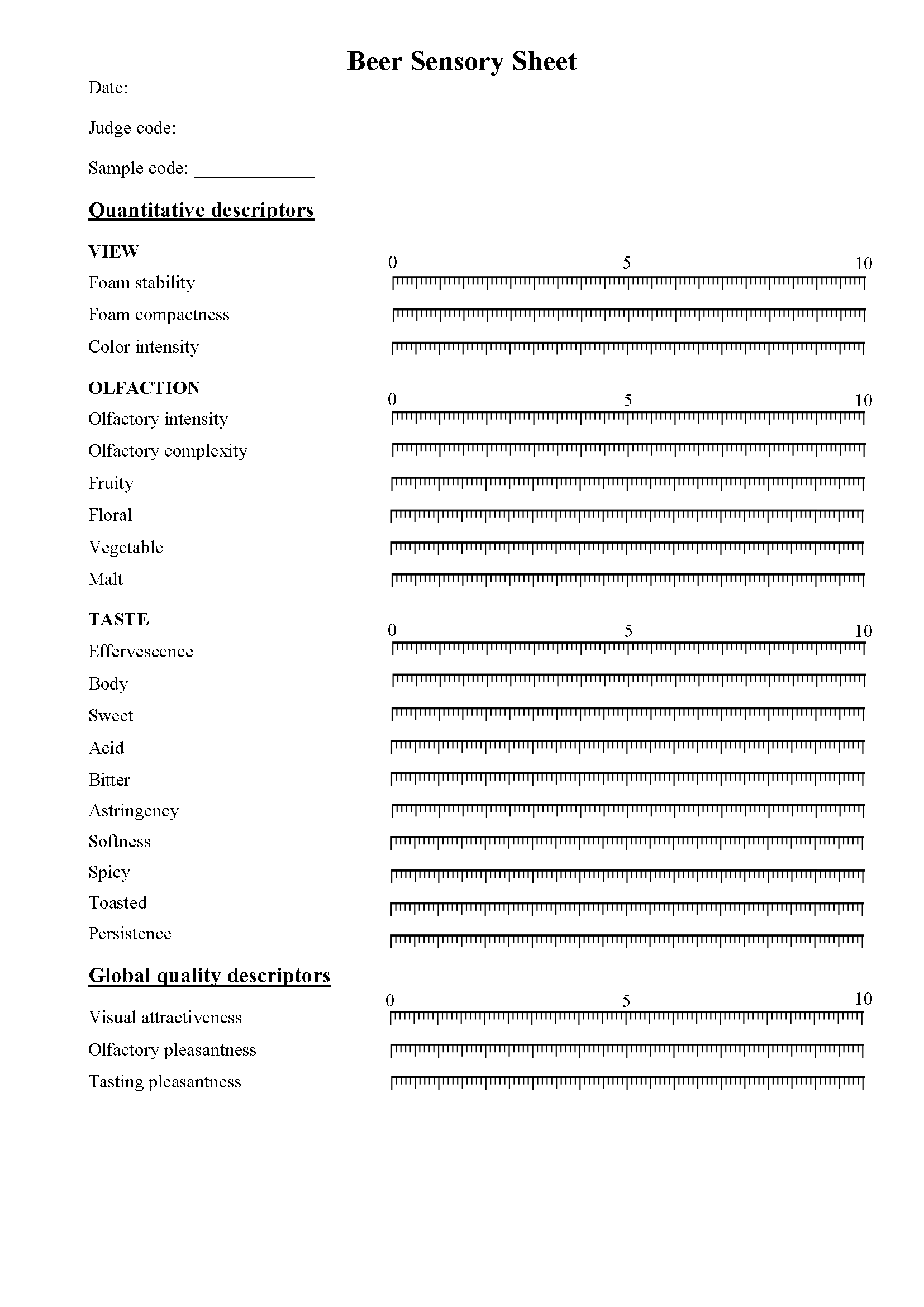


**Figure S1.** Sensory sheet used by the panel for the sensory evaluation of beers.
